# Use of a Port-a-Cath to Facilitate ECT, a Novel UK Case

**DOI:** 10.1192/bjo.2026.11765

**Published:** 2026-06-30

**Authors:** Ozan Aydin

**Affiliations:** Swansea Bay UHB, Swansea, United Kingdom

## Abstract

**Aims:**

Electroconvulsive therapy (ECT) remains the most effective and potentially life-saving treatment for severe psychotic depression, particularly in older adults with treatment-resistant illness and high suicide risk. However, delivery of ECT may be rendered impossible by repeated failure of peripheral venous access, leading to premature discontinuation of an otherwise effective intervention. This case aims to highlight the ethical, clinical and practical considerations of using a totally implantable venous access device (Port-a-Cath) solely to enable continuation of ECT in an exceptional, high-risk psychiatric context.

**Methods:**

We report the case of a 76-year-old woman with recurrent, severe psychotic depression and baseline mild cognitive impairment, known to psychiatric services for over six years with multiple admissions under the Mental Health Act. She had a clear and consistent historical response to ECT, including resolution of psychotic symptoms and recovery of cognitive function to baseline. During a prolonged relapse marked by nihilistic somatic delusions, profound functional impairment and persistent suicidal ideation requiring constant 1:1 observations, ECT was recommenced but had to be abandoned due to repeated failure of peripheral venous access. Alternative strategies, including ultrasound-guided cannulation, midline and PICC lines, were deemed unsuitable or unsustainable. Following multidisciplinary discussion, a Port-a-Cath was inserted to enable reliable anaesthetic access and continuation of ECT.

**Results:**

The Port-a-Cath was inserted without complication and successfully facilitated recommencement of ECT after a short healing period. Subsequent treatments were delivered reliably, with good-quality seizures and no device-related adverse events. Over the course of ECT, the patient demonstrated gradual but meaningful improvement in psychomotor retardation, thought blocking, affective reactivity and somatic delusions, alongside a reduction in suicidal ideation. Cognitive functioning improved in parallel with mood, consistent with her prior illness trajectory. The device remained functional and unobtrusive, reducing repeated procedural distress and eliminating risks associated with repeated cannulation in a highly vulnerable patient lacking capacity to consent.

**Conclusion:**

This case demonstrates that, in exceptional circumstances, a Port-a-Cath may be a safe, ethical and effective means of enabling access to life-saving ECT when standard venous access options have failed. Careful multidisciplinary decision-making, proportionality and ongoing risk–benefit evaluation are essential. Innovative approaches to treatment delivery may be justified to uphold patient dignity and maximise recovery in severe, otherwise intractable psychiatric illness.

